# Enhancing waste management awareness in future nurses: a theory-based educational intervention and psychometric evaluation of a measurement tool

**DOI:** 10.1136/bmjopen-2026-116465

**Published:** 2026-08-06

**Authors:** Neslihan Soylemez, Hilal Yıldırım

**Affiliations:** 1 Osmaniye Korkut Ata Universitesi, Osmaniye, Turkey; 2 Inonu Universitesi, Malatya, Turkey

**Keywords:** PUBLIC HEALTH, Nurses, Health Education, Awareness, Psychometrics

## Abstract

**Objective:**

This study was conducted to evaluate the effect of waste separation and recycling education on waste management awareness among nursing students at a university and to examine the psychometric properties of a measurement tool designed to assess this awareness.

**Method:**

The research was conducted using a single-group pretest–post-test quasi-experimental and methodological design. The population of the study consisted of individuals receiving nursing education; 180 participants were included in the pretest and 117 in the post-test. Data were collected using a Demographic Information Form and the Waste Management Awareness Scale. Following the pretest, a three-session, theory-based educational intervention focused on waste separation and recycling, each lasting approximately 40 min, was implemented and the post-test was administered after the third session.

**Findings:**

The average age of participants was 20.37±1.52 and the majority were female (79.4%). The two-subscale structure of the scale was psychometrically supported and explained 58.20% of the total variance. A significant increase was found in waste management awareness scores after the training (p<0.001). The training intervention was found to have a moderate effect on waste management awareness and waste separation behaviours and a small-to-moderate effect on attitudes towards recycling (Cohen’s d=0.30–0.44).

**Conclusion:**

The findings show that nursing students have a moderate level of waste management awareness and that theory-based structured education significantly strengthens this awareness. It is recommended that waste management awareness, as a measurable and developable construct, be addressed systematically and sustainably within nursing education.

**STRENGTHS AND LIMITATIONS OF THIS STUDY**

- The study combined a quasi-experimental design with psychometric validation procedures.
- Scale validity was evaluated using expert review, exploratory factor analysis and confirmatory factor analysis.
- The educational intervention was delivered using a standardised educational protocol.
- The study was conducted at a single university using a quasi-experimental design without a control group.

## Introduction

 Waste management awareness is a multidimensional structure encompassing individuals’ knowledge levels regarding waste types, their attitudes towards the environmental and economic benefits of recycling and the behaviours they exhibit in this regard.^1^ The increase in waste volume on a global scale and the fact that a significant portion of health-related waste is recyclable has made effective waste management dependent not only on technical infrastructure but also on the level of awareness among individuals.^1 2^ This situation demonstrates that waste management awareness is a critical issue not only for the environment but also for public health and sustainability.^3^

Studies in the literature show that although attitudes towards recycling (ATC) and waste separation are generally positive, these attitudes are not sufficiently reflected in behaviour.^4^ Although the vast majority of individuals believe that recycling is necessary, it has been reported that the proportion of those who regularly separate waste remains limited.^3 4^ Similarly, research in the health field shows that although healthcare workers and students have sufficient knowledge about waste management, inconsistencies occur in the separation of recyclable waste.^5 6^

In the Turkish context, waste management awareness is more limited due to certain structural and cultural barriers.^5 7^ Only a small portion of municipal waste in Turkey can be included in the recycling process.^3 4^ Studies conducted with university students in Turkey reveal that recycling awareness is generally moderate, but there are serious difficulties in translating this awareness into action.^8 9^ The perception that individual contributions to recycling are insignificant and that environmental responsibility is largely attributed to institutions are among the main reasons for these difficulties.^7^

Nursing students are a group that takes active responsibility in waste management processes due to their professional roles and sets an example for patients, their relatives and society through their behaviour.^10^ Although waste management topics are included in different courses in nursing education, it is observed that recycling and zero waste practices are not sufficiently reinforced at the behavioural level due to the intensive curriculum and clinical priorities.^11^ This situation may limit the development of sustainable waste management behaviours in postgraduation practices.^10 11^

It is noteworthy that a significant portion of studies on waste management use questionnaires that have not been tested for validity and reliability.^8 12^ Measurement tools with undefined psychometric properties make it difficult to objectively evaluate the accuracy of the findings and the effectiveness of educational programmes. This situation indicates a significant gap in the development of a valid and reliable Waste Management Awareness Scale (WMAS) that encompasses both attitude and behaviour dimensions.

### Rationale and theoretical basis for the educational intervention

Education-based interventions are among the most effective strategies in the literature for developing waste management awareness.^13 14^ It has been reported that structured education programmes, especially those implemented for university students and groups studying in the health field, strengthen ATC, increase environmental awareness and bring about significant improvements in separation behaviours.^5 10 11^ These findings indicate that waste management awareness is a construct that can be developed not only through information but also through targeted educational interventions.^14^

International studies have demonstrated that educational interventions can be effective at the behavioural level.^9 13 15^ A study conducted with university students in Thailand found an increase of over 25% in correct waste separation behaviours following structured recycling education.^16^ Similarly, university-based intervention studies conducted in Spain and Italy showed that short-term but interactive training had a significant impact on students’ environmental attitudes and sustainable behaviour intentions.^17–19^ In Turkey, however, educational interventions on waste management are limited and mostly remain at the information level.^3 8 9 20^ Some studies conducted with university students indicate that the training increased knowledge levels but did not provide sufficient evidence regarding the sustainability of behavioural change.^21 22^ This situation highlights the need for structured, behaviour-focused training programmes for nursing students.

The Theory of Planned Behavior (TPB) provides a strong theoretical basis for this need. According to TPB, the emergence of behaviour is shaped by the individual’s attitude toward the behaviour, perceived social norms and perceived control over performing the behaviour.^23^ The educational intervention applied in this study aimed to provide information about waste types, develop positive attitudes towards the environmental and social benefits of recycling, and strengthen students’ perceptions of self-efficacy regarding their ability to sustain these behaviours in their daily lives. The use of interactive methods in the education process activates the cognitive and affective components that support behavioural intention as predicted by TPB.^14 23^ In this context, education was considered not only as a teaching activity in this study but also as a fundamental intervention component aimed at demonstrating that waste management awareness is a structure that can be measurably changed.^23 24^ Accordingly, the aim of this study is to evaluate the effect of waste separation and recycling education for nursing students on waste management awareness within the framework of the TPB and to develop the WMAS to measure this awareness.

In this context, the research seeks to answer the following questions:

1. Does the waste separation and recycling education provided to nursing students increase their level of waste management awareness?
2. Is the developed WMAS a valid and reliable tool for measuring nursing students’ attitudes and behaviours regarding waste management?

## Method

### Research design

This study employed a single-group pretest–post-test quasi-experimental design to evaluate the effectiveness of the educational intervention and a cross-sectional methodological design to evaluate the psychometric properties of the WMAS. The psychometric analyses (content validity, exploratory factor analysis (EFA), confirmatory factor analysis (CFA) and internal consistency) were performed using baseline (pretest) data collected before the intervention.

The reporting of this study followed the Strengthening the Reporting of Observational Studies in Epidemiology (STROBE) Statement for cross-sectional studies to improve the transparency and completeness of reporting.

### Location and time of the study

The research was conducted at a university in eastern Türkiye during the Spring 2025 Academic Year.

### Population and sample

The study population consisted of 986 undergraduate nursing students enrolled in a 4-year nursing programme at the study university during the 2025 academic year. The minimum required sample size was determined using power analysis for a single-group pretest–post-test experimental design. Assuming a 95% confidence level, 80% statistical power and an effect size of 0.50, at least 105 participants were required.

Students were informed about the study during scheduled class sessions and were invited to participate voluntarily. A total of 212 students agreed to participate and completed the baseline assessment. The study sample was then selected using simple random sampling from among these volunteers. Student lists were prepared, and participants were selected using a computer-generated random selection procedure. To enable matching of pretest and post-test data, each participant was assigned a unique identification code.

Following data screening, 32 questionnaires were excluded because of incomplete responses. Consequently, 180 participants were included in the psychometric analyses, including EFA, CFA and reliability analyses. After completion of the educational intervention, 117 participants completed the post-test assessment and were included in the intervention effectiveness analyses.

Eligibility criteria were: (1) being enrolled as an undergraduate nursing student at the study university; (2) being aged 18 years or older; (3) providing written informed consent; (4) volunteering to participate; and (5) completing the baseline questionnaire. Participants with incomplete questionnaires were excluded from the psychometric analyses. For evaluation of the educational intervention, only participants who attended all educational sessions and completed both the pretest and post-test assessments were included.

### Data collection process and tools

Data were collected through face-to-face administration using a student introduction form and the WMAS developed by the researchers. The student introduction form collected baseline demographic and educational characteristics, including age, sex, previous training on hospital waste management, previous training on waste separation and recycling and participants’ baseline knowledge regarding selected waste management practices (eg, disposal of medical waste, expired medications and recyclable waste). The WMAS was administered immediately before and after the educational intervention to evaluate changes in waste management awareness. Completion of all data collection instruments required approximately 15 min.

### Development of scale items

Scale items related to waste management awareness and recycling were developed following a comprehensive review of the relevant literature addressing environmental sustainability, waste management practices and nursing education.^10 11 19 22 24^ In addition, item development was guided by the TPB, which proposes that health-related behaviours are influenced by attitudes, perceived behavioural control and behavioural intentions.^23^ Accordingly, the initial item pool was designed to capture both behavioural practices related to waste separation and recycling and attitudes toward environmentally responsible waste management. A comprehensive item pool was initially generated and subsequently formatted using a five-point Likert-type response scale.^25^

### Content and face validity

After the scale items related to waste management awareness were created, expert opinion was sought to evaluate the relevance, clarity and comprehensiveness of each item.^26^ A panel of four experts (one in public health nursing, one in nursing fundamentals and two in surgical nursing), all holding doctoral degrees, independently evaluated each item using a four-point relevance scale (1=not appropriate, 2=needs major revision, 3=needs minor revision and 4=appropriate). The Content Validity Index (CVI) was calculated based on expert ratings, and the overall Scale CVI was 0.90, indicating excellent content validity.

Based on expert recommendations, conceptually overlapping items were merged, items considered outside the intended construct were removed and wording revisions were made to improve clarity and comprehensibility. As a result, the initial item pool was reduced from 39 to 18 items before psychometric testing.

Subsequently, EFA, CFA and reliability analyses were performed. Items with inadequate psychometric performance (eg, low factor loadings or poor contribution to internal consistency) were removed, resulting in the final nine-item WMAS.

### Construct validity and internal consistency

Construct validity of the WMAS was evaluated using EFA followed by CFA. Before factor extraction, sampling adequacy was assessed using the Kaiser–Meyer–Olkin (KMO) coefficient and Bartlett’s test of sphericity. Internal consistency reliability was evaluated using Cronbach’s alpha coefficients and corrected item–total correlations.^27 28^

### Exploratory factor analysis

EFA was conducted using principal component analysis with Varimax rotation to identify the underlying factor structure of the WMAS. Before factor extraction, sampling adequacy was assessed using the KMO coefficient and Bartlett’s test of sphericity. A KMO value ≥0.60 and a statistically significant Bartlett’s test (p<0.05) were considered indicative of suitability for factor analysis.^27 29^ Factors with eigenvalues greater than 1.0 were retained. Items with factor loadings ≥0.40 and cross-loading differences of at least 0.10 were considered acceptable for retention. The resulting factor structure was interpreted in accordance with the TPB, with particular attention to the conceptual distinction between behavioural practices and attitudes toward waste management.^23 26 29^

### Confirmatory factor analysis

To further evaluate the construct validity of the WMAS, a CFA was conducted based on the two-factor structure identified in the EFA. The model demonstrated an acceptable-to-good fit to the data (χ²/df=2.36, Goodness-of-Fit Index (GFI)=0.937, Adjusted Goodness-of-Fit Index (AGFI)=0.886, Standardized Root Mean Square Residual (SRMR)=0.064, Incremental Fit Index (IFI)=0.943, Tucker–Lewis Index (TLI)=0.917, Comparative Fit Index (CFI)=0.942, Root Mean Square Error of Approximation (RMSEA)=0.087). These findings support the proposed two-factor structure of the WMAS, indicating that the model provides an adequate representation of the observed data.

**Figure 1:**
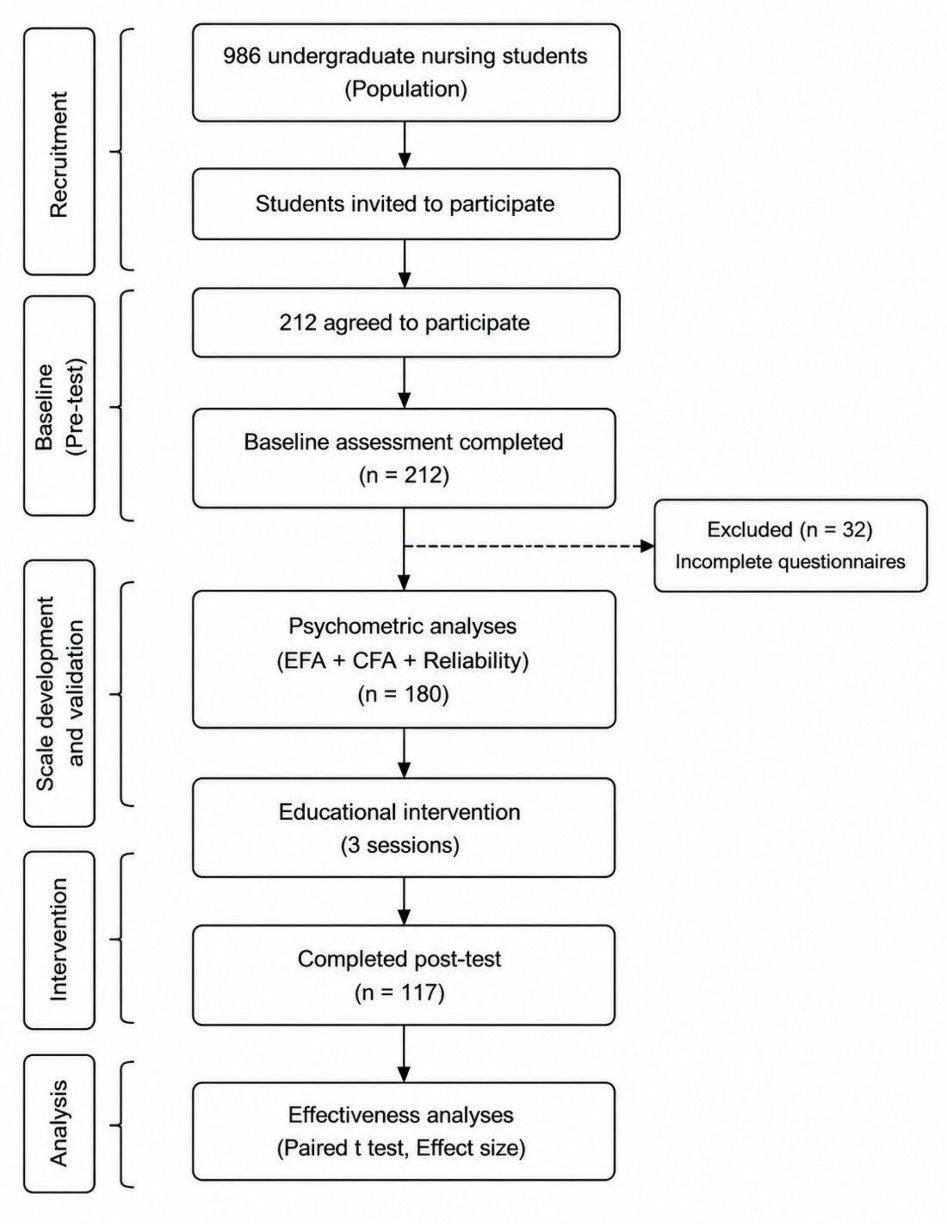
Flow diagram of participant recruitment, sample selection, psychometric validation process and educational intervention. CFA, confirmatory factor analysis; EFA, exploratory factor analysis.

### Second-order confirmatory factor analysis

To further examine whether the two first-order factors reflected an overarching construct of waste management awareness, a second-order CFA was performed. The second-order model demonstrated acceptable-to-good model fit (χ²/df=2.36, GFI=0.937, AGFI=0.886, SRMR=0.069, IFI=0.943, TLI=0.917, CFI=0.942, RMSEA=0.087). These findings indicate that the two-factor structure can be adequately represented by a higher-order latent construct, providing additional evidence for the structural validity of the WMAS.

The first-order and second-order CFA models are presented in online supplemental figures S1 and S2, respectively, together with the corresponding model fit indices (online supplemental tables S1 and S2).

### Waste Management Awareness Scale

The WMAS was developed to determine the behaviour and attitude levels of nursing students regarding waste management. The scale was designed as a measurement tool to assess the awareness of individuals, particularly those educated in the health field, regarding environmental sustainability, waste separation and recycling. However, the WMAS can also be used in different sample groups to evaluate the effectiveness of education and awareness programmes.

The final form of the scale consists of a total of nine items and has a two-subscale structure. The scale items cover the behaviours individuals exhibit towards waste management in their daily lives and during the provision of health services, as well as their ATC. The WMAS is rated on a 5-point Likert scale (5=Always, 4=Most of the time, 3=Sometimes, 2=Rarely, 1=Never). The total score that can be obtained from the scale ranges from 9 to 45. An increase in the total score obtained from the scale indicates an increase in individuals’ awareness of waste management.

Waste Separation and Recycling Behaviour (WSRB): This first subdimension evaluates individuals’ daily behaviours regarding waste separation and recycling practices for glass, paper, packaging, plant waste, batteries and similar waste. This subdimension contains a total of five items, ranging from one to five. The score that can be obtained from the subdimension varies between 5 and 25. A high score indicates that individuals’ WSRBs are positive and adequate.

ATC: This second subdimension evaluates individuals’ attitudes and beliefs regarding the usefulness and environmental effects of recycling. The subdimension contains a total of four items, ranging from six to nine. The score that can be obtained from the subdimension varies between 4 and 20. High scores obtained from this subdimension indicate that individuals have positive ATC.

No cut-off has been determined for the WMAS. There are no items that need to be reversed. The scale was developed not for diagnostic purposes but to assess individuals’ waste management awareness as a continuous variable. The total score that can be obtained from the scale ranges from 9 to 45 points. As the scores obtained from the scale and its subscales increase, it is assumed that individuals’ behaviours and attitudes towards waste management are more positive.

### Educational intervention

As part of the research, a structured training programme covering waste separation, recycling and zero waste was implemented for nursing students. The training programme aimed not only to provide information about waste generated in the healthcare sector but also to raise awareness among young individuals about recycling and environmental sustainability in their daily lives. The training content included: (1) types of waste generated in healthcare services (medical, hazardous, recyclable and household waste), (2) basic recycling and zero waste principles, (3) the environmental impacts of recyclable products (eg, rubber, textile and packaging waste), (4) proper waste separation and appropriate disposal methods, and (5) the roles and responsibilities of nurses and young individuals in waste management. The educational content is structured within the framework of the TPB to strengthen students’ attitudes toward waste management, increase their perception of individual responsibility that supports recycling behaviours and support perceived behavioural control that they can apply these behaviours in their daily lives.^13 16 23 24^

The training sessions were planned during the students’ free time, taking their class schedules into account and were conducted in groups of 10–12 students. The training sessions were conducted in a quiet meeting room with visual presentations to support attention and interaction. In addition to lectures, brainstorming, case study discussions and feedback-based interactive methods were used during the training process. These approaches were chosen to encourage active student participation and support the translation of acquired knowledge into behaviour.

The training programme was implemented in three sessions, each lasting approximately 40 min. The pretest was administered 1 week before the training programme, and the post-test was administered 1 week after the completion of the training. This time frame was determined to allow students to internalise the information they had acquired and to evaluate possible behavioural changes.

The educational intervention was delivered by the principal researcher, a faculty member in public health nursing with experience in environmental health, nursing education and educational intervention research. To ensure consistency, all training sessions were delivered by the same instructor using a standardised educational protocol, identical presentation materials and the same interactive teaching methods.

#### Patient and public involvement

Patients and/or the public were not involved in the design, conduct, reporting or dissemination plans of this research.

### Statistical analysis

Statistical analyses were performed using IBM SPSS Statistics V.26.0 (IBM, Armonk, New York, USA). CFA was conducted using IBM SPSS AMOS V.24.0. Descriptive statistics were presented as frequencies, percentages, means and SD. EFA was applied to assess the construct validity of the scale; sample adequacy was tested using the KMO coefficient and Bartlett’s sphericity test. The internal consistency of the scale was assessed using Cronbach’s alpha coefficient and item-total correlations.

To determine the effectiveness of the educational intervention, pretest and post-test scores were compared using a paired samples t-test. The effect size was calculated using Cohen’s d coefficient. The level of statistical significance was set at p<0.05.

## Findings

Table 1 describes the participants’ characteristics. The mean age of the participants was 20.37±1.52, and 79.4% were female. During their nursing education, 80% received training on hospital waste management (medical waste, sharps waste). 44.4% received training on waste separation and recycling. 66.7% stated that the tissue used to wipe the patient’s hand is medical waste because it came into contact with the patient. 26.1% stated that expired medications should be disposed of in household waste bins. 42.2% of participants stated that a large portion of hospital waste consists of household waste and that this waste can be collected with municipal waste.

**Table 1:** Descriptive characteristics of nursing students (n=180)

Characteristics		N	%
Gender			
Female		143	79.4
Male		37	20.6
Receive training on hospital waste management (medical, sharps waste) during nursing education.			
Yes		144	80
No		36	20
Receive training on waste separation and recycling.			
Yes		80	44.4
No		100	55.6
The tissue used to wipe the patient’s hand is medical waste because it came into contact with the patient**.**			
Yes		120	66.7
No		60	33.3
Expired medications should be disposed of in household waste bins.			
Yes		47	26.1
No		133	73.9
A large portion of hospital waste consists of household waste, which can be collected with municipal waste.			
Yes		76	42.2
No		104	57.8
	Min	Max	Mean±SD
Age	19	35	20.37±1.52

Baseline demographic characteristics and baseline WMAS scores did not differ significantly between participants who completed the post-test assessment and those who were lost to follow-up (all p>0.05), suggesting that attrition was unlikely to have introduced substantial selection bias.

In the assessment conducted prior to EFA in table 2, the KMO value was found to be 0.836 and the Bartlett sphericity test result χ², p=0.000 was found to be statistically significant. These results indicate that the data are suitable for factor analysis.

**Table 2:** Maximum likelihood analysis followed by varimax rotation, factor loadings and item-total correlations of the items of the WMAS scale (n=180)

Scale items	Mean (SD)	Corrected item-total correlation	Cronbach’s alpha if item deleted	Factor loading	Alpha	Variance
Waste Sorting and Recycling Behaviours	**16.26** (4.25)				0.828	**32.33**
I take care to collect batteries separately.	3.61 (0.99)	0.596	0.821	0.538		
I collect paper and packaging waste separately; I do not throw it in the trash can.	3.29 (1.02)	0.685	0.810	0.721		
I collect glass waste separately; I do not throw it in the trash can.	3.37 (1.09)	0.760	0.800	0.771		
I collect plant waste (vegetables, apricot pits, peanut shells, etc) separately; I do not throw it in the trash can.	2.88 (1.21)	0.547	0.828	0.830		
I collect small amounts of waste, such as frying oil, separately and do not throw them in the trash.	3.11 (1.18)	0.540	0.829	0.741		
Attitude Towards Recycling	**15.23** (2.33)				0.680	**25.87**
I do not allow any material to be used unnecessarily.	3.89 (0.77)	0.443	0.836	0.529		
Recycling generates financial gain.	4.17 (0.71)	0.382	0.841	0.739		
I purchase products that have a recycling symbol on them.	3.39 (0.91)	0.376	0.843	0.673		
I throw non-recyclable waste in the trash and recyclable waste in the recycling bins.	3.78 (0.84)	0.695	0.813	0.742		
Total	**26.21** (5.05)				0.842	**58.20**

Bold values indicate the summary statistics (subscale and total scale mean scores, Cronbach's alpha coefficients and percentage of explained variance) rather than statistically significant values.

WMAS, Waste Management Awareness Scale.

EFA revealed that the scale has a two-factor structure. The total variance explained by the factors is 58.20%, and the WMAS total mean is 26.21±5.05, indicating a moderate level. EFA revealed that the scale has a two-subscale structure. The first subdimension is named ‘Waste Separation and Recycling Behaviors’, consists of five items and explains 32.33% of the total variance (Cronbach’s α=0.828), with a WSRB mean score of 16.26±4.25. The second subdimension is named ‘Attitudes Towards Recycling’, consists of four items and explains 25.87% of the total variance (Cronbach’s α=0.680), with a mean score of 15.23±2.33. The total Cronbach’s alpha coefficient of the scale is 0.842, which is considered highly reliable. The suitability of the scale items is determined by items with factor loadings of 0.30 and above; in this study, the factor loadings of the scale items were above 0.50.

Table 3 shows the preintervention and postintervention activity evaluation of participants’ waste management awareness. A significant increase was observed in the total score of the WMAS after the training (pretest: 31.60±5.64; post-test: 34.65±5.64; mean difference=3.05; t(116)=4.78; p=0.006), and the effect size was moderate (Cohen’s d=0.44). In the subscale analyses, WSRB scores increased significantly in the post-test (pretest: 16.29±4.17; post-test: 18.53±3.85; mean difference=2.23; t(116)=4.81; p=0.020; d=0.44). ATC scores also showed a significant increase after the training (pretest: 15.30±2.03; post-test: 16.11±2.33; mean difference=0.81; t(116)=3.28; p=0.006) and the effect size was small to medium (d=0.30).

**Table 3:** The comparison of pretest and post-test mean scores in the WMAS

Variable		Experimental Group (N: 117) M±SD	Mean difference (Post–pre)	T (df)	P value	Cohen’s d	95% CI
	Pretest	31.60±5,64					
**WMAS**			3.05	4.78 (116)	**0.006**	0.44	4.31 to 1.78
	Post-test	34.65±5.64					
	Pretest	16.29±4.17					
**WSRB**			2.23	4.81 (116)	**0.020**	0.44	3.16 to 1.31
	Post-test	18.53±3.85					
	Pretest	15.30±2.03					
**ATC**			0.81	3.28 (116)	**0.006**	0.30	1.30 to 0.32
	Post-test	16.11±2.33					

Paired-samples t-test. Statistically significant results (p<0.05) are in bold.

ATC, Attitude Towards Recycling; WMAS, Waste Management Awareness Scale; WSRB, Waste Separation and Recycling Behaviour.

## Discussion

The descriptive findings obtained in this study indicate that although participants reported having acquired basic knowledge about hospital waste management to a large extent, they experienced significant conceptual confusion in their knowledge and assessments of waste separation and recycling practices. It is noteworthy that while 80% of participants stated that they received training on medical waste and sharps waste management during their nursing education, only 44.4% reported receiving training on waste separation and recycling. This suggests that waste management in the nursing curriculum is addressed more in the context of infection control and medical waste safety, while recycling and zero waste components remain relatively limited. It should also be considered that approximately 44% of participants had previously received training related to waste separation and recycling before the intervention. Previous exposure to waste management concepts may have influenced baseline awareness levels and may have contributed to the magnitude of the observed intervention effect. Nevertheless, despite this previous exposure, significant improvements were still observed following the structured educational programme, suggesting that theory-based education provides additional educational benefit beyond prior knowledge.

The fact that 66.7% of students classified tissues used to wipe the patient’s hands as medical waste on the grounds that they came into contact with the patient, and 26.1% stated that expired medications should be disposed of in household waste bins, reveals gaps in knowledge and incorrect generalisations regarding the classification of waste types. Similarly, 42.2% of participants stating that household waste constitutes a large portion of hospital waste and that this waste can be collected together with municipal waste indicates that the risk-based separation principle in waste management has not been sufficiently internalised. These findings reveal that students may develop excessive risk perceptions in some cases, such as *considering hand wipes as medical waste*, while in other cases, they may normalise practices that are risky in terms of environmental and public health, such as *disposing of medications as household waste*.

The literature contains studies reporting similar inconsistencies in knowledge, attitudes and behaviours among nursing students and healthcare workers.^6 11 15^ International studies show that although healthcare workers have a high level of knowledge about the management of medical waste, there are significant shortcomings in the separation of recyclable waste and the proper disposal of medication waste. Studies conducted in Turkey also report that nursing students and healthcare workers have a moderate level of knowledge regarding waste management, and that behavioural consistency cannot be achieved, particularly in recycling and zero waste practices.^21 30 31^ In our study, participants’ awareness of waste management and their recycling behaviours and attitudes were moderate. When evaluated together with descriptive data, these findings reveal that nursing students are familiar with the basic concepts of waste management but have difficulty translating this knowledge into consistent and correct behaviours. Within the framework of the TPB, this situation suggests that positive attitudes and knowledge levels alone are not sufficient for behavioural change; perceived behavioural control and guidance for implementation play a critical role.^23^

In this context, the structured educational intervention applied in the study aimed not only to increase nursing students’ knowledge levels but also to correct false generalisations and conceptual errors and to reinforce attitudes that support recycling and waste separation behaviours. Such misperceptions that emerged prior to the training clearly demonstrate why structured and behaviour-focused training is necessary. Furthermore, evaluating this process with a valid and reliable measurement tool is important in order to objectively demonstrate the effectiveness of the training.

### Effect of the educational intervention

This study found that the structured educational intervention led to a moderate improvement in waste management awareness and sorting behaviour, and a small to moderate improvement in ATC. The increase observed in the total score for waste management awareness after the training suggests that the intervention created a comprehensive effect targeting not only knowledge but also attitude and behaviour components. The moderate effect size obtained demonstrates that a short-term, group-based education programme can create meaningful change even in a topic such as waste management, which is often considered secondary.

When evaluated at the subdimension level, it is noteworthy that the increase in scores related to WSRBs is more pronounced than that in the attitude dimension. This indicates that structured and application-oriented training has a faster and more direct impact, particularly on behavioural components. The literature reports that environmental education interventions can have stronger effects on the behavioural dimension, while attitudes are more influenced by cultural norms, habits and social context.^1 5 7 32^ The small-to-moderate effect observed in the attitude dimension in this study supports the notion that attitudes are more resistant and shaped over time compared with behaviours.^10 15^

International studies report that interactive and structured recycling education programmes for university students increase behavioural gains, but attitude change remains more limited.^10 18 21 24^ In the Turkish context, the limited perception of the individual contribution to recycling and the evaluation of environmental responsibility at the institutional level may cause attitude change to occur more slowly.^7 12 20^ This cultural context may explain why the effect size in the attitude dimension was found to be lower than that in the behaviour dimension in this study.

When evaluated within the framework of the TPB, these findings suggest that the educational intervention strengthened behavioural intention by supporting students’ attitudes toward waste management and their perceived behavioural control levels. The significant differences found between premeasurements and postmeasurements indicate that structured education reveals that waste management awareness is a developable construct. In this regard, the first research question of the study, ‘Does the waste separation and recycling education provided to nursing students increase their level of waste management awareness?’, is supported.

### Structural characteristics of the scale

The two-factor structure of the WMAS developed in this study supports the idea that waste management awareness is a multidimensional structure consisting of both behavioural and attitudinal components. Similarly, studies in the literature examining environmental awareness and recycling behaviours treat behaviour and attitude as separate but interrelated dimensions.^6 15 19^ This indicates that waste management awareness cannot be explained solely by knowledge or positive attitudes; practices that translate into behaviour must be evaluated as a separate dimension.^22^

The structuring of the first subdimension of the scale as ‘Waste Separation and Recycling Behaviors’ is important in terms of reflecting the behaviours of nursing students that can be directly observed in daily life and professional practice.^32 33^ The high internal consistency of this subdimension suggests that behavioural items are perceived more clearly and consistently by students. Indeed, some studies in the health field have reported that recycling behaviours exhibit a more homogeneous structure compared with attitudes.^6 11 15^ This finding is consistent with the more pronounced increase in the post-training behaviour dimension obtained in the study.

The naming of the second subdimension as ‘Attitude Towards Recycling’ is meaningful in that it reflects students’ perceptions of the benefits of recycling, its necessity and the importance of individual contribution. The fact that the internal consistency coefficient in this dimension is lower than that in the behavioural dimension may be due to attitudes exhibiting a more variable and context-sensitive structure compared with behaviours.^14 24^ The literature indicates that environmental attitudes are strongly influenced by cultural norms, social expectations and individual experiences and may therefore exhibit more heterogeneous structures.^7 16 23^ This may explain why the attitude dimension shows lower but acceptable levels of reliability.

The fact that the total variance of the scale is above 50% and the items have high factor loadings indicates that the developed measurement tool presents a conceptually consistent structure.^28 29^ Considering that some scales developed in the field of environmental awareness and recycling report total explained variance in the range of 40–60%, the construct validity results obtained in this study appear consistent with the literature.^26 27 29^ Furthermore, the high total internal consistency coefficient of the scale indicates that it provides a reliable measurement that addresses both attitude and behaviour dimensions simultaneously.^27^

When evaluated together with the descriptive results of the study, these findings reveal that although nursing students possess knowledge about waste management, they experience differences in their attitudes and behaviours. The developed scale makes this difference visible, enabling the identification of pretraining deficiencies and the objective evaluation of post-training change. Considering the limited availability of measurement tools in Turkey that have been tested for validity and reliability in the field of waste management and that address both attitude and behaviour dimensions, this scale is thought to make an important contribution to both nursing education and public health research. In line with these findings, the second research question of the study, ‘Does the Waste Management Awareness Scale validly and reliably measure nursing students’ attitudes and behaviors regarding waste management?’, is answered positively. The fact that the two-factor structure of the scale is theoretically meaningful, that the items are consistently grouped under the relevant dimensions, and that the internal consistency coefficients are above acceptable levels, indicates that the developed measurement tool is a valid and reliable tool that can be used in both research and educational applications.

## Limitations

This study has some limitations. First, the research was conducted using a single-group quasi-experimental pretest–post-test design. The main reason for not using a control group was that knowledge sharing among students in the same academic environment is inevitable and may blur the intervention effect, posing a risk of contamination. This approach is also used as an ethical and methodological choice in similar studies evaluating education-based interventions.

The fact that the study sample consisted of nursing students studying at a single university limits the generalisability of the findings. Since waste management awareness is a context-sensitive construct that can be influenced by cultural norms, national recycling policies and infrastructure capabilities, the results obtained in different societies and populations may vary. Therefore, the use of the scale in different populations is recommended. The relatively short duration of the educational intervention and the fact that measurements were only taken in the short term limit the ability to comment on the sustainability of behavioural change. Future studies are recommended to use designs that include supportive reminder activities and longitudinal follow-up assessments. Furthermore, the fact that the scale was developed and the effectiveness of the training was evaluated using the same sample requires careful interpretation in terms of measurement sensitivity. Validating studies using different samples will strengthen both the psychometric power of the scale and the generalisability of the training interventions.

Reasons for loss to follow-up were not systematically collected during the study. Therefore, the potential influence of attrition on the intervention findings should be interpreted with caution.

In addition, test–retest reliability of the WMAS could not be evaluated because the psychometric validation was performed using baseline cross-sectional data collected before the intervention. Furthermore, criterion-related (convergent) validity was not assessed because no previously validated instrument measuring the same construct was available in the study context. Future studies should evaluate both temporal stability and convergent validity using independent samples.

Although no significant baseline differences were observed between participants who completed the post-test and those lost to follow-up, the possibility of residual attrition bias cannot be completely excluded.

## Supplementary material

10.1136/bmjopen-2026-116465 online supplemental file 1

10.1136/bmjopen-2026-116465 online supplemental file 2

10.1136/bmjopen-2026-116465 online supplemental file 3

## Data Availability

Data are available upon reasonable request.
