# Supplementary material for: Enhancing waste management awareness in future nurses: a theory-based educational intervention and psychometric evaluation of a measurement tool
Source: BMJ Open. 2026 Aug 6;16(8):e116465. doi: 10.1136/bmjopen-2026-116465 (PMC13448624; doi:10.1136/bmjopen-2026-116465)
Supplement: Supplementary file 1 [file bmjopen-2026-116465.reviewer_comments.pdf]

PEER REVIEW HISTORY

BMJ Open publishes all reviews undertaken for accepted manuscripts. Reviewers are asked to complete a checklist review form and are provided with free text boxes to elaborate on their assessment. These free text comments are reproduced below.

ARTICLE DETAILS

Title (Provisional)

Enhancing Waste Management Awareness in Future Nurses: A Theory-Based Educational Intervention and Psychometric Evaluation of a Measurement Tool

Authors

SOYLEMEZ, Neslihan; Yıldırım, Hilal

VERSION 1 - REVIEW

**Reviewer**	**1**
**Name**	**Mukaihata, Tsuyoshi**
**Affiliation**	**Hyogo Medical University, Department of Nursing School**
**Date**	**15-Feb-2026**
**COI**	**None**

Thank you for the opportunity to review this manuscript.

1. Design: Please consider clearly stating the study design used to evaluate the psychometric properties of the scale (e.g., cross-sectional or longitudinal).

2. Reporting guidelines: If any reporting guidelines (e.g., STROBE, CONSORT, or TREND) were used to structure the manuscript, please state this explicitly, as it would strengthen the methodological transparency of the study.

3. Population and sample: Please describe the eligibility criteria for participant inclusion.

4. Please clearly report (1) the number of students who were invited to participate in the study and (2) the number of students who agreed to participate.

5. Regarding the statement, “The sample was selected using simple random sampling from among the students who agreed to participate in the study” (p.5), please report how many students were randomly selected.

6. Regarding the statement that experts evaluated each item for content validity and clarity (p.6), the evaluation procedure is not sufficiently described. Please clarify how content validity was assessed (e.g., rating criteria, use of a content validity index) and how decisions to retain, revise, or remove items were made.

7. The descriptions of construct validity (p.6) and exploratory factor analysis (p.7) appear to overlap substantially. Please consider consolidating these sections to reduce redundancy and improve clarity.

8. Regarding the statement, "The factor structure obtained as a result of the exploratory factor analysis was evaluated by considering the theoretical framework and item contents" (p.7), please consider explaining the theoretical framework more clearly here or earlier in the manuscript (e.g., in the Introduction or the section on Development of Scale Items).

9. Details regarding who delivered the intervention should be reported in the Educational Program section. Please clarify who delivered the educational program (e.g., researchers, nursing faculty, or other professionals).

10. In the Methods section, the baseline data collected in addition to the WMAS are not clearly specified. Please describe the demographic and other baseline variables assessed (e.g., age, sex, prior training related to waste management) to improve transparency and reproducibility.

11. Please specify the statistical software used for the analyses (including the software name and version) in the analysis section.

12. Given the substantial attrition from pre-test to post-test, it would be helpful to report the reasons for loss to follow-up, if such information is available, in the Methods or Results section. If these data were not collected, please acknowledge this as a limitation.

13. Given the substantial attrition from pre-test to post-test, please compare baseline characteristics (e.g., demographics and baseline WMAS scores) between participants who completed the post-test and those who were lost to follow-up, and report any differences in the Methods or Results section. If such comparisons were not conducted, please acknowledge this as a limitation.

14. Given that some participants had prior training related to waste management/recycling at baseline, please discuss in the Discussion or Limitations section how this prior training may have influenced the observed intervention effects.

15. Please acknowledge in the Limitations section that the test–retest reliability of the WMAS could not be evaluated due to the cross-sectional design of the scale development, and that criterion-related (or convergent) validity was not assessed through comparison with existing validated measures.

**Reviewer**	**2**
**Name**	**Xie, Shiqi**
**Affiliation**	**Chongqing Medical University, Nursing School**
**Date**	**29-Jun-2026**

COI **None**

1.The study uses a single-group pretest-posttest quasi-experimental design without a control group, which may compromise internal validity due to history or maturation effects. Please elaborate on the rationale for not including a control group and discuss the limitations regarding causal inference.

2.There is a notable dropout rate (from 180 to 117, ~35%). Discuss the reasons for attrition and whether it may bias the results.

3.The intervention consists of only three 40-minute sessions. Consider adding longer-term follow-up (e.g., 3–6 months) to assess the sustainability of behavioral changes.

4.While EFA supports a two-factor structure, no CFA was conducted. We recommend validating the factor structure in an independent sample.

5.The Cronbach's α for the "Attitude" subscale is 0.68, slightly below the conventional threshold of 0.70. Discuss the acceptability of this reliability and consider revising or adding items.

6.The scale lacks interpretative cutoffs or categories (e.g., low/moderate/high awareness). Providing percentile-based benchmarks would enhance practical utility.

7.Multiple paired t-tests were conducted without adjustment for multiplicity. Consider applying Bonferroni correction to control Type I error.

8.Effect sizes (Cohen's d = 0.30–0.44) are reported but without confidence intervals. Including CIs would improve interpretability.

9.Some paragraphs in the Methods section are lengthy and could benefit from clearer subheadings and logical flow.

VERSION 1 - AUTHOR RESPONSE

Reviewer: 1

Dr. Tsuyoshi Mukaihata, Hyogo Medical University.

1. *Design: Please consider clearly stating the study design used to evaluate the psychometric properties of the scale (e.g., cross-sectional or longitudinal).*

Response: Thank you for this valuable suggestion. We have clarified the study design in the Methods section. The educational intervention was conducted using a single-group pretest–posttest quasi-experimental design, whereas the psychometric evaluation of the WMAS was

performed using a cross-sectional methodological design based on baseline (pre-test) data collected before the intervention.

2. *Reporting guidelines: If any reporting guidelines (e.g., STROBE, CONSORT, or TREND) were used to structure the manuscript, please state this explicitly, as it would strengthen the methodological transparency of the study.*

Response: Thank you for this valuable suggestion. The manuscript has been revised in accordance with the STROBE (Strengthening the Reporting of Observational Studies in Epidemiology) Statement to improve the transparency and completeness of reporting. In addition, the completed STROBE checklist has been provided as a Supplementary File for the Editor and reviewers.

3. *Population and sample: Please describe the eligibility criteria for participant inclusion.*

Response: Thank you for this valuable suggestion. We have revised like that: "Eligibility criteria included: (1) being enrolled as an undergraduate nursing student at the study university, (2) being aged 18 years or older, (3) voluntarily agreeing to participate, (4) attending all educational sessions, (5) completing both the pre-test and post-test assessments, and (6) completing all questionnaire items. Students who provided incomplete questionnaires or did not attend all intervention sessions were excluded from the final analyses."

4. *Please clearly report (1) the number of students who were invited to participate in the study and (2) the number of students who agreed to participate.*

Response: Thank you for this important suggestion. We have revised the Population and Sample section to clearly report the participant recruitment process. The study population consisted of 986 undergraduate nursing students. All eligible students were invited to participate during scheduled class sessions, and 212 students voluntarily agreed to participate and completed the baseline assessment. In addition, a participant flow diagram has been added (Figure 1) to improve the transparency of the recruitment and study process.

5. *Regarding the statement, "The sample was selected using simple random sampling from*

among the students who agreed to participate in the study" (p.5), please report how many students were randomly selected.

Response: Thank you for this valuable comment. We have clarified the sampling procedure in the revised manuscript. **Simple random sampling was performed among the 212 students who voluntarily agreed to participate.** After data screening, **32 questionnaires were excluded because of incomplete responses**, leaving **180 participants** for the psychometric analyses. The sampling procedure and participant flow are now described in greater detail in the *Population and Sample* section and illustrated in **Figure 1**.

6. *Regarding the statement that experts evaluated each item for content validity and clarity (p.6), the evaluation procedure is not sufficiently described. Please clarify how content validity was assessed (e.g., rating criteria, use of a content validity index) and how decisions to retain, revise, or remove items were made.*

Response: Thank you for this valuable comment. We have substantially expanded the description of the content validity procedure in the Methods section. Specifically, we now report the composition of the expert panel, the four-point rating scale used for item evaluation (1 = not appropriate to 4 = appropriate), the calculation of the Content Validity Index (S-CVI = 0.90), and the criteria used to revise or remove items. We also clarified that conceptually overlapping items were merged, items outside the intended construct were removed following expert review, and additional items were eliminated after psychometric analyses (EFA, CFA, and reliability analyses), resulting in the final nine-item scale.

7. *The descriptions of construct validity (p.6) and exploratory factor analysis (p.7) appear to overlap substantially. Please consider consolidating these sections to reduce redundancy and improve clarity.*

Response: Thank you for this helpful observation. We agree that there was unnecessary overlap between the descriptions of construct validity and exploratory factor analysis. To improve readability, these sections have been revised and reorganized. General information regarding construct validity and reliability is now presented under the "Construct Validity and Internal Consistency" heading, whereas methodological details specific to the exploratory factor analysis are described only once under the "Exploratory Factor Analysis" subsection.

This revision has substantially reduced redundancy and improved the overall clarity of the Methods section.

8. *Regarding the statement, "The factor structure obtained as a result of the exploratory factor analysis was evaluated by considering the theoretical framework and item contents" (p.7), please consider explaining the theoretical framework more clearly here or earlier in the manuscript (e.g., in the Introduction or the section on Development of Scale Items).*

Response: Thank you for this insightful suggestion. We have clarified the theoretical framework underlying the development of the WMAS. Specifically, the *Development of Scale Items* section has been expanded to explain that the item pool was developed based on both the relevant literature and the **Theory of Planned Behavior (TPB)**, which guided the inclusion of items assessing attitudes and behavioral practices related to waste management. In addition, the *Exploratory Factor Analysis* section has been revised to clarify that interpretation of the final factor structure was informed by the TPB framework and the conceptual relevance of the retained items.

9. *Details regarding who delivered the intervention should be reported in the Educational Program section. Please clarify who delivered the educational program (e.g., researchers, nursing faculty, or other professionals).*

Response: Thank you for this valuable suggestion. We have clarified who delivered the educational intervention. The manuscript now states that all educational sessions were delivered by the principal researcher, a faculty member in Public Health Nursing with experience in environmental health and educational interventions. We also clarified that the same instructor, standardized presentation materials, and identical educational protocol were used throughout all sessions to ensure consistency of the intervention.

10. *In the Methods section, the baseline data collected in addition to the WMAS are not clearly specified. Please describe the demographic and other baseline variables assessed (e.g., age, sex, prior training related to waste management) to improve transparency and reproducibility.*

Response: Thank you for this helpful comment. We have expanded the *Data Collection Process and Tools* section to clearly describe all baseline variables collected in addition to the

WMAS. Specifically, we now report that the Student Introduction Form included demographic variables (age and sex), previous training related to hospital waste management and recycling, and baseline knowledge regarding selected waste management practices. This revision improves the transparency and reproducibility of the study.

11. *Please specify the statistical software used for the analyses (including the software name and version) in the analysis section.*

Response: Thank you for this suggestion. The Statistical Analysis section has been revised to specify the statistical software used. IBM SPSS Statistics version 26.0 was used for all statistical analyses, and IBM SPSS AMOS version 24.0 was used for the confirmatory factor analysis.

12. *Given the substantial attrition from pre-test to post-test, it would be helpful to report the reasons for loss to follow-up, if such information is available, in the Methods or Results section. If these data were not collected, please acknowledge this as a limitation.*

Response: Thank you for this important observation. Unfortunately, reasons for loss to follow-up were not systematically recorded during data collection. We have therefore acknowledged this issue as a limitation of the study in the revised manuscript.

13. *Given the substantial attrition from pre-test to post-test, please compare baseline characteristics (e.g., demographics and baseline WMAS scores) between participants who completed the post-test and those who were lost to follow-up, and report any differences in the Methods or Results section. If such comparisons were not conducted, please acknowledge this as a limitation.*

Response: Thank you for this valuable suggestion. We compared the baseline demographic characteristics and baseline WMAS scores of participants who completed the post-test with those who were lost to follow-up. No statistically significant differences were observed (all $p > 0.05$), suggesting that attrition was unlikely to have introduced substantial selection bias. This information has been added to the Results section. Nevertheless, because the reasons for loss to follow-up were not systematically recorded, the possibility of residual attrition bias cannot be completely excluded and has been acknowledged as a study limitation.

14. *Given that some participants had prior training related to waste management/recycling at baseline, please discuss in the Discussion or Limitations section how this prior training may have influenced the observed intervention effects.*

Response: Thank you for this insightful comment. We have expanded the Discussion section to acknowledge that some participants had received previous training related to waste management and recycling before the intervention. We now discuss how this prior exposure may have influenced baseline awareness and the observed intervention effects.

15. *Please acknowledge in the Limitations section that the test–retest reliability of the WMAS could not be evaluated due to the cross-sectional design of the scale development, and that criterion-related (or convergent) validity was not assessed through comparison with existing validated measures.*

Response: Thank you for this valuable suggestion. We have revised the Limitations section to acknowledge that test–retest reliability and criterion-related (convergent) validity were not evaluated in the present study. We also recommend that future studies examine these psychometric properties using independent samples.

Reviewer: 2

Dr. Shiqi Xie, Chongqing Medical University

1.*The study uses a single-group pretest-posttest quasi-experimental design without a control group, which may compromise internal validity due to history or maturation effects. Please elaborate on the rationale for not including a control group and discuss the limitations regarding causal inference.*

Response: Thank you for this important comment. We agree that the absence of a control group limits causal inference. As recommended, we have expanded the Limitations section to clarify the rationale for using a single-group design. Because students studied in the same educational environment, contamination between intervention and control groups was considered

unavoidable. We also explicitly acknowledged that this design limits causal inference and recommended controlled studies in future research.

__2.__ There is a notable dropout rate (from 180 to 117, ~35%). Discuss the reasons for attrition and whether it may bias the results.

Response: Thank you for this important comment. We agree that participant attrition should be carefully considered when interpreting intervention studies. Although the reasons for loss to follow-up were not systematically recorded, we compared the baseline characteristics of participants who completed the post-test with those who were lost to follow-up. No statistically significant differences were observed in demographic characteristics or baseline WMAS scores (all $p > 0.05$), suggesting that attrition was unlikely to have introduced substantial selection bias. This information has been added to the Results section, and the absence of systematically collected reasons for attrition has been acknowledged as a study limitation.

These findings suggest that the observed attrition was unlikely to have materially influenced the intervention results.

__3.__The intervention consists of only three 40-minute sessions. Consider adding longer-term follow-up (e.g., 3–6 months) to assess the sustainability of behavioral changes.

Response: Thank you for this valuable suggestion. We agree that longer follow-up assessments would provide stronger evidence regarding the sustainability of behavioral change. The present study was designed to evaluate the short-term effectiveness of the educational intervention. We have acknowledged this limitation in the manuscript and recommended that future studies include longer follow-up periods (e.g., 3–6 months) to evaluate the long-term sustainability of waste management awareness and behaviors.

__4.__While EFA supports a two-factor structure, no CFA was conducted. We recommend validating the factor structure in an independent sample.

Response: Thank you for this valuable suggestion. In response, we conducted a confirmatory factor analysis (CFA) using AMOS to validate the two-factor structure identified in the exploratory factor analysis. The CFA demonstrated acceptable-to-good model fit ($\chi^2/df=2.36$,

GFI=0.937, AGFI=0.886, CFI=0.942, TLI=0.917, IFI=0.943, RMSEA=0.087). The CFA model and fit indices have been added to the manuscript and are also presented as supplementary material.

Analyzing both EFA and CFA within the same sample is a scientifically acceptable and validated approach in modern psychometrics, as these methods are complementary frameworks derived from the same underlying factor model **(Widaman & Helm, 2023**). Furthermore, recent methodological advancements, such as Exploratory Structural Equation Modeling (ESEM), demonstrate that combining exploratory and confirmatory elements within a single sample provides a statistically robust and realistic representation of complex constructs (**Marsh et al., 2014)."**

Marsh, H. W., Morin, A. J., Parker, P. D., & Kaur, G. (2014). Exploratory structural equation modeling: An integration of the best features of exploratory and confirmatory factor analysis. Annual review of clinical psychology, 10(1), 85-110.

Widaman, K. F., & Helm, J. L. (2023). Exploratory factor analysis and confirmatory factor analysis. In H. Cooper, M. N. Coutanche, L. M. McMullen, A. T. Panter, D. Rindskopf, & K. J. Sher (Eds.), APA handbook of research methods in psychology: Data analysis and research publication (2nd ed., pp. 379–410). American Psychological Association.

This approach has also been adopted in previous psychometric studies.

Yildirim, H., Isik, K., & Cengizhan, S. Ö. (2021). Psychometric evaluation of the healthy aging instrument in older adults. *Educational Gerontology*, *47*(6), 235-246.

Yildirim, H., & Işik, K. (2021). Psychometric evaluation of the Hospitalisation-Related Stressors Questionnaire for Elderly Patients. Psychogeriatrics, 21(2), 166-174.

In addition, to provide further evidence for the hierarchical structure of the WMAS, we also conducted a second-order confirmatory factor analysis. The second-order model demonstrated acceptable-to-good model fit indices, and the consistency of the findings across both first-order and second-order CFA provides additional support for the construct validity of the scale. The second-order CFA model and its fit indices have been included as supplementary material.

5.*The Cronbach's α for the "Attitude" subscale is 0.68, slightly below the conventional threshold of 0.70. Discuss the acceptability of this reliability and consider revising or adding items.*

Response: We thank the reviewer for this comment. Although the Cronbach's alpha coefficient of the Attitude subscale was 0.68, values between 0.60 and 0.70 are considered acceptable for newly developed scales, particularly when the number of items is limited. Because this subscale contains only four items and demonstrated satisfactory factor loadings and construct validity, no additional items were added in order to preserve the conceptual structure of the scale.

Values between 0.60 and 0.70 are generally considered acceptable for newly developed scales and exploratory research **(Hair et al., 2019; Furr, 2021).**

Hair Jr, J. F., Black, W. C., & Babin, B. J. (2019). Multivariate Data Analysis, 8/E.

Furr, R. M. (2021). Psychometrics: an introduction. SAGE publications.

6.*The scale lacks interpretative cutoffs or categories (e.g., low/moderate/high awareness). Providing percentile-based benchmarks would enhance practical utility.*

Response: We appreciate this suggestion. However, the WMAS was developed as a continuous psychometric measure of awareness rather than as a diagnostic instrument. Therefore, establishing cut-off scores was beyond the scope of the present study. Interpretation should be based on continuous total and subscale scores rather than categorical classifications. This clarification has been added to the manuscript.

Furthermore, transforming continuous psychometric scores into categorical classifications using arbitrary cut-off points is strongly discouraged in statistical methodology, as it leads to a substantial loss of information, reduction in statistical power, and increased risk of Type II errors (**Cohen, 1983; MacCallum et al., 2002; Royston et al., 2006**). Since the WMAS captures a dimensional construct rather than a categorical clinical diagnosis, treating and analyzing the scores on a continuum is psychometrically more justified and robust.

Cohen, J. (1983). The cost of dichotomization. *Applied psychological measurement*, *7*(3), 249-253.

MacCallum, R. C., Zhang, S., Preacher, K. J., & Rucker, D. D. (2002). On the practice of dichotomization of quantitative variables. *Psychological methods*, *7*(1), 19.

Royston, P., Altman, D. G., & Sauerbrei, W. (2006). Dichotomizing continuous predictors in multiple regression: a bad idea. Statistics in medicine, 25(1), 127-141.

7.*Multiple paired t-tests were conducted without adjustment for multiplicity. Consider applying Bonferroni correction to control Type I error*.

Response: We thank the reviewer for this suggestion. Bonferroni correction is generally recommended when conducting multiple exploratory comparisons. In the present study, however, the paired comparisons were limited to the total score and the two predefined subscales specified before data analysis. Therefore, adjustment for multiplicity was not applied, consistent with recommendations for confirmatory analyses of prespecified outcomes.

Adjustment for multiplicity is not always recommended for prespecified confirmatory analyses because it may unnecessarily increase Type II error **(Rothman, 1990).**

Rothman, K. J. (1990). No adjustments are needed for multiple comparisons. Epidemiology, 1(1), 43-46.

8*.Effect sizes (Cohen's d = 0.30–0.44) are reported but without confidence intervals. Including CIs would improve interpretability.*

Response: Thank you for this suggestion. We agree that confidence intervals around effect sizes may improve interpretation. However, because Cohen's d was reported as a supplementary measure of intervention magnitude and the paired t-test results are already accompanied by 95% confidence intervals for the mean differences, we retained the original presentation.

Reporting Cohen's d alongside 95% confidence intervals (CIs) for mean differences strictly aligns with contemporary reporting standards. Methodologists emphasize that CIs capture the precision of unstandardized effects, while standardized metrics like Cohen's d complementarily illustrate the practical magnitude of the intervention (**Cumming, 2014; Durlak, 2009**). This dual approach satisfies the recommendations of the American Psychological Association (**APA, 2020**) for transparent reporting.

American Psychological Association. (2020). Publication manual of the American psychological association 2020. *American Psychological Association*.

Durlak, J. A. (2009). How to select, calculate, and interpret effect sizes. *Journal of pediatric psychology*, *34*(9), 917-928.

Cumming G. (2014). The new statistics: why and how. Psychological Science, 25(1), 7-29.

***9**.Some paragraphs in the Methods section are lengthy and could benefit from clearer subheadings and logical flow.*

Response:. Thank you for this valuable suggestion. The Methods section has been reorganized with clearer subheadings and improved logical flow to enhance readability. Because this study includes both psychometric scale development and evaluation of an educational intervention, the methodology is necessarily comprehensive. To improve clarity, we reorganized the Methods section using clearly defined subheadings, including Research Design, Population and Sample, Development of Scale Items, Content and Face Validity, Construct Validity and Internal Consistency, Exploratory Factor Analysis, Confirmatory Factor Analysis, Educational Intervention, Data Collection Process and Tools, and Statistical Analysis. This revision provides a more logical, transparent, and reader-friendly presentation of the study procedures.

We sincerely appreciate the reviewers' thoughtful and constructive comments. Their suggestions substantially improved the methodological rigor, transparency, and overall quality of our manuscript. We hope that the revised version satisfactorily addresses all concerns and will now be considered suitable for publication in BMJ Open.

VERSION 2 - REVIEW

**Reviewer**	**1**
**Name**	**Mukaihata, Tsuyoshi**
**Affiliation**	**Hyogo Medical University, Department of Nursing School**
**Date**	**26-Jul-2026**
**COI**	

Thank you for the opportunity to review this manuscript again. I have no further comments.

**Reviewer**	**2**

Name Xie, Shiqi

Affiliation Chongqing Medical University, Nursing School

Date 28-Jul-2026

COI

Revised as requested. Accepted for publication.
