# Supplementary material for: Enhancing waste management awareness in future nurses: a theory-based educational intervention and psychometric evaluation of a measurement tool
Source: BMJ Open. 2026 Aug 6;16(8):e116465. doi: 10.1136/bmjopen-2026-116465 (PMC13448624; doi:10.1136/bmjopen-2026-116465)
Supplement: Supplementary file 2 [file bmjopen-16-8-s001.docx]

**Figure 1.** First-order confirmatory factor analysis (CFA) model of the Waste Management Awareness Scale (WMAS).

**Table 4.** Confirmatory Factor Analysis Fit Indices for the Waste Management Awareness Scale (WMAS)

| **Fit Index** | **Recommended Value** | **Obtained Value** | **Interpretation** |
| --- | --- | --- | --- |
| χ²/df | <3.00 | 2.364 | Good fit |
| GFI | >0.90 | 0.937 | Good fit |
| AGFI | >0.85 | 0.886 | Good fit |
| SRMR | <0.08 | 0.064 | Good fit |
| IFI | >0.90 | 0.943 | Good fit |
| TLI | >0.90 | 0.917 | Good fit |
| CFI | >0.90 | 0.942 | Good fit |
| RMSEA | <0.08 ideal; <0.10 acceptable | 0.087 | Acceptable fit |

**Confirmatory Factor Analysis**

To further evaluate the construct validity of the Waste Management Awareness Scale (WMAS), a confirmatory factor analysis (CFA) was conducted based on the two-factor structure identified in the exploratory factor analysis. The model demonstrated an acceptable-to-good fit to the data (χ²/df = 2.36, GFI = 0.937, AGFI = 0.886, SRMR = 0.064, IFI = 0.943, TLI = 0.917, CFI = 0.942, RMSEA = 0.087). These findings support the proposed two-factor structure of the WMAS, indicating that the model provides an adequate representation of the observed data.
