# Supplementary material for: Enhancing waste management awareness in future nurses: a theory-based educational intervention and psychometric evaluation of a measurement tool
Source: BMJ Open. 2026 Aug 6;16(8):e116465. doi: 10.1136/bmjopen-2026-116465 (PMC13448624; doi:10.1136/bmjopen-2026-116465)
Supplement: Supplementary file 4 [file bmjopen-16-8-s003.docx]

**Tittle Page**

**Enhancing Waste Management Awareness in Future Nurses: A Theory-Based Educational Intervention and Psychometric Evaluation of a Measurement Tool**

^*^Neslihan SOYLEMEZ ^**^Hilal YILDIRIM

*Neslihan SOYLEMEZ(Phd) Research Assistant, Department of Surgical Nursing, Faculty of Health Sciences, Osmaniye Korkut Ata University, Osmaniye, Turkey/ 80000 ORCID ID: 0000-0001-6137-3634 mail: [neslihansoylemez@osmaniye.edu.tr](mailto:neslihansoylemez@osmaniye.edu.tr) Tel: +90-328-827-10-00

c

**(Correspondent Writer) Hilal YILDIRIM (Phd), Assoc. Prof. Dr, Department of Public Health Nursing, Faculty of Nursing, Inonu University, Malatya, Turkey/44000, ORCID ID: 0000-0003-3971-3799 E-mail: [firat.hilal@inonu.edu.tr](mailto:firat.hilal@inonu.edu.tr) Tel: +90-422-377 30 60 /1271 Fax: +90-422-341 02 19

ETHICS

Before starting the study, Ethical approval was obtained from the Inonu University Health Sciences Scientific Research Ethics Committee (approval no. 2023/5431).

ACKNOWLEDGMENTS

The authors gratefully acknowledge the nursing students who participated in this study and contributed to the successful completion of the research.

COMPETİNG INTERESTS

Hilal Yıldırım is affiliated with Inonu University, which provided financial support for this study. The funder had no role in the design of the study, data collection, analysis, interpretation of the data, manuscript preparation, or the decision to publish. All other authors declare no competing interests.

FUNDING

This research was supported by the Scientific Research Projects Unit (BAPSİS) of İnönü University under project number TSA-2025-3978.

DATA AVAILABILITY STATEMENT

Data available on request from the authors.

INFORMED CONSENT:

Informed consent was obtained from all individual participants included in the study.

AUTHOR CONTRIBUTIONS

Neslihan Soylemez and Hilal Yıldırım: Conceptualization, Formal analysis, Investigation, Resources, Original Draft, Writing – Review & Editing, Visualization, Supervision.
